# Electroacupuncture Attenuates CFA-induced Inflammatory Pain by suppressing Nav1.8 through S100B, TRPV1, Opioid, and Adenosine Pathways in Mice

**DOI:** 10.1038/srep42531

**Published:** 2017-02-13

**Authors:** Hsien-Yin Liao, Ching-Liang Hsieh, Chun-Ping Huang, Yi-Wen Lin

**Affiliations:** 1 College of Chinese Medicine, Graduate Institute of Acupuncture Science, China Medical University, Taichung 40402, Taiwan; 2 Department of Acupuncture, China Medical University Hospital, Taichung 40402, Taiwan; 3 College of Chinese Medicine, Graduate Institute of Integrated Medicine, China Medical University, Taichung 40402, Taiwan; 4 Department of Chinese Medicine, China Medical University Hospital, Taichung 40402, Taiwan; 5 Research Center for Chinese Medicine & Acupuncture, China Medical University, Taichung 40402, Taiwan; 6 Department of Life Sciences, National Chung Hsing University, Taichung 40401, Taiwan; 7 College of Chinese Medicine, School of Post-Baccalaureate Chinese Medicine, China Medical University, Taichung 40402, Taiwan; 8 College of Chinese Medicine, Master’s Program for Traditional Chinese Veterinary Medicine, China Medical University, Taichung 40402, Taiwan.

## Abstract

Pain is associated with several conditions, such as inflammation, that result from altered peripheral nerve properties. Electroacupuncture (EA) is a common Chinese clinical medical technology used for pain management. Using an inflammatory pain mouse model, we investigated the effects of EA on the regulation of neurons, microglia, and related molecules. Complete Freund’s adjuvant (CFA) injections produced a significant mechanical and thermal hyperalgesia that was reversed by EA or a transient receptor potential V1 (TRPV1) gene deletion. The expression of the astrocytic marker glial fibrillary acidic protein (GFAP), the microglial marker Iba-1, S100B, receptor for advanced glycation end-products (RAGE), TRPV1, and other related molecules was dramatically increased in the dorsal root ganglion (DRG) and spinal cord dorsal horn (SCDH) of CFA-treated mice. This effect was reversed by EA and TRPV1 gene deletion. In addition, endomorphin (EM) and N^6^-cyclopentyladenosine (CPA) administration reliably reduced mechanical and thermal hyperalgesia, thereby suggesting the involvement of opioid and adenosine receptors. Furthermore, blocking of opioid and adenosine A1 receptors reversed the analgesic effects of EA. Our study illustrates the substantial therapeutic effects of EA against inflammatory pain and provides a novel and detailed mechanism underlying EA-mediated analgesia via neuronal and non-neuronal pathways.

Inflammatory pain can result from thermal, chemical, or mechanical injuries via nociceptors in the neural system^1^. Inflammation-associated changes typically cause hypersensitization to the chemical environment surrounding nerve fibers^1^. Damaged cells release endogenous factors that activate nerve fibers and neighboring non-neural cells (e.g., astrocytes, microglia, platelets, and immune cells). Nociceptive neuron sensitivity is modulated by several inflammatory mediators in the extracellular environment. Investigations into the cellular components involved in this process have greatly enhanced our understanding of nociceptive mechanisms and facilitated attempts to cure pain. An inflammatory state can be created by injecting chemical agents, such as complete Freund’s adjuvant (CFA) or carrageenan, into model systems^2^. The induced inflammatory pain travels upstream to the spine and cortical brain regions via action potentials, channels, receptors, and signaling molecules. The central nervous system comprises approximately 100 billion neurons and 10-fold more glial cells^3^.

Several channels, receptors, and signaling molecules within neurons and microglia are responsible for pain transmission. Secreted by astrocytes, S100-B is often implicated in the central nervous system (CNS)^4^. S100-B proteins then activate receptors for advanced glycation end-products (RAGE), which results in acute and chronic diseases^5^. RAGE activation initiates downstream inflammatory cellular responses^6^, and increased levels of RAGE have been reported in neurons and glia after brain injury^7^. The Nav sodium channels are involved in inflammation-induced hyperalgesia^8^,^9^. Sodium channel-induced currents that significantly influence the threshold for action potential firing have been identified in neurons of the CNS^9^ and DRG^8^. Ion channel transient receptor potential vanilloid 1 (TRPV1) plays an important role in both nociceptive^10^ and neuropathic pain^11^. TRPV1 is expressed in peripheral dorsal root ganglion (DRG), central spinal cord dorsal horn (SCDH), and brain. Centrally expressed TRPV1 is involved in the detection of thermal and mechanical pain^12^. The PI3K/AKT/mTOR (mTORC1) signaling pathway is involved in cellular immunity^13^. In addition, the activation of TRPV1 increases the expression of PI3K, AKT, CREB, NF-κB, Nav1.7, and Nav1.8. The increased expression of these molecules was attenuated in TRPV1^−/−^ mice^12^.

Acupuncture has been used for over 3,000 years in Asia to treat pain, and the analgesic efficacy of acupuncture is recognized worldwide. Over the past thirty years, studies have investigated the relationship between acupuncture and endogenous central opiates^14^. However, relatively recent studies showed that the antinociceptive effect of acupuncture may be related to changes in the expression of various ionotropic receptor channels and voltage-gated channels, including N-methyl-D-aspartate receptors (NMDARs), acid-sensing ion channel 3 (ASIC3), TRPV1, local adenosine, and Nav channels^12^,^15^,^16^,^17^,^18^. Our previous studies demonstrated that EA results in antinociceptive effects and reduces mechanical and thermal hyperalgesia in an inflammatory mouse model via inhibition of TRPV1 and its related pathways^12^. However, the complete mechanism behind the effects of EA on neurons and microglia remains unclear. Thus, we assessed the expression of non-neuronal markers, including GFAP, Iba-1, S100B, and RAGE, and neuronal TRPV1-related molecules during inflammatory pain. This study provides new information on the relationships between EA, inflammatory pain, neurons, and microglia.

## Material and Methods

### Experimental Animals

All animals were treated in accordance with the National Institute of Health Guide for the Care and Use of Laboratory Animals, and the study protocol was approved by the ethics committee of the China Medical University, Taichung, Taiwan (permit No. 2016-061). C57/B6 mice weighing approximately g and aged 8–12 weeks were purchased from the BioLASCO Animal Center, Taipei, Taiwan. Animals were housed in Plexiglas cages in a temperature-controlled room (25 ± 2 °C) with a relative humidity of 60 ± 5%, and were fed a diet of standard rat chow and water ad libitum. Approximately hours before the experiment, the rats were fasted but had free access to water.

### Inflammatory Pain Model

Based on our previous studies^12^, a total of ten mice per group was the minimum number necessary for fully powered experiments. The mice were subdivided randomly into four groups of: (1) Control group: normal saline injection, (2) CFA group: CFA injection to induce inflammatory pain, (3) EA group: CFA injection and EA manipulation, and (4) TRPV1^−/−^ group: CFA injection to determine the role of TRPV1 in inflammatory pain. All experiments were performed in the laboratory during daylight hours. Two EA sessions were completed at 24 hours and 48 hours after CFA injection between 9:00 and 10:00 am. We used behavioral testing and the Hargreaves test to assess mechanical and thermal hyperalgesia at baseline, the moment after injection of CFA, and 24 and 48 hours after CFA injection. Two days after the CFA injection and EA sessions, we compared the pain reducing effects of EA and TRPV1 gene knockout. We analyzed pain-related molecules in the DRG and SCDH of mice using western blotting and immunohistochemical staining. Mice were anesthetized with 1% isoflurane and injected with 20 μl of saline (pH 7.4, buffered with 20 mM HEPES) or CFA (complete Freund’s adjuvant; 0.5 mg/ml heat-killed *M. tuberculosis*; Sigma, St. Louis, MO) in the plantar surface of the hind paw to induce intraplantar inflammation.

### Electroacupuncture

EA was applied using stainless steel needles (0.5″ inch, 32 G, YU KUANG, Taiwan) that were inserted into the muscle layer to a depth of 2–3 mm at ST36 acupoint. EA was administered 1 day after the CFA injection every day at the same time (10:00–12:00 AM). A Trio-300 (Japan) stimulator delivered electrical square pulses for 15 min with a 100 μs duration and a 2 Hz frequency. The stimulation amplitude was 1 mA.

### Behavior Test (von Frey test and Hargraves’ test)

Behavior tests were conducted at 1–2 day after induction of CFA injection. All stimuli were performed at room temperature (approximately 25 °C) and applied only when the animals were calm but not sleeping or grooming. Mechanical sensitivity was measured by testing the force of responses to stimulation with three applications of electronic von Frey filaments (North Coast Medical, Gilroy, CA, USA). Thermal pain was measured with three applications using Hargraves’ test IITC analgesiometer (IITC Life Sciences, SERIES8, Model 390 G).

### Opioid or adenosine A1 receptor agonist and antagonist administration

Adult C57BL/6 male mice (n = 10) aged 8 to 12 weeks were used in this experiment. Twenty-four hours after inflammation was induced as described above, the μ opioid agonist endomorphin (EM) (Sigma, St. Louis, MO, USA), in 100 μl of saline, was administered intraperitoneally (i.p.) at a dose of 10 mg/kg once a day. Alternatively, the adenosine receptor agonist N^6^-cyclopentyladenosine (CPA) (Sigma, St. Louis, MO, USA) in 10 μl of saline was administered intramuscularly (i.m.) at a dose of 0.1 mg/kg into acupoint ST36 once a day under light isoflurane anesthesia (1%). The opioid antagonist naloxone methiodide (Nal) (Sigma, St. Louis, MO, USA) in 100 μl of saline was injected i.p. at a dose of 10 mg/kg. The adenosine A1 receptor antagonist rolofylline (Ro) (Sigma, St. Louis, MO, USA) in 10 μl of saline was injected i.m. at a dose of 3 mg/kg into acupoint ST36. The PI3K inhibitor LY294002 (2.5 μg/10 μl, Sigma, St. Louis, MO, USA) was dissolved in 10% DMSO and injected into acupoint ST36 (10 μl). The vehicle control group received an injection of 10 μl 10% DMSO.

### Tissue sampling, Western Blot, and Immunohistochemical staining

Mice aged 8–12 weeks were killed by use of CO2 to minimize their suffering. L3-L5 DRG and SCDH were harvested on day 2 after CFA injection and then immediately excised to extract proteins. Total proteins were prepared by homogenized DRG and SCDH in lysis buffer containing 50 mM Tris-HCl pH 7.4, 250 mM NaCl, 1% NP-40, 5 mM EDTA, 50 mM NaF, 1 mM Na3VO4, 0.02% NaN3 and 1× protease inhibitor cocktail (AMRESCO). The extracted proteins (30 μg per sample assessed by BCA protein assay) were subjected to 8% SDS-Tris glycine gel electrophoresis and transferred to a PVDF membrane. The membrane was blocked with 5% nonfat milk in TBS-T buffer (10 mM Tris pH 7.5, 100 mM NaCl, 0.1% Tween 20), incubated with first antibody (anti-iba-1, anti-pp38, anti-s100B, anti-RAGE, anti-TRPV1, anti-pPI3K, anti-pAKT, anti-pmTOR, anti-pCREB, anti-pNF-κB, anti-Nav1.7, anti-Nav1.8, anti-COX-2, anti-pPKCε) in TBS-T with 1% bovine serum albumin, and incubated for 1 hour at room temperature. Peroxidase-conjugated anti-rabbit antibody (1:5000) was used as a secondary antibody. The bands were visualized by an enhanced chemiluminescencent substrate kit (PIERCE) with LAS-3000 Fujifilm (Fuji Photo Film Co. Ltd). Where applicable, the image intensities of specific bands were quantified with NIH ImageJ software (Bethesda, MD, USA). The stained DRG and SCDH slices were sealed under the coverslips, and then examined for the presence of immune-positive DRG and SCDH neurons using an epifluorescent microscope (Olympus, BX-51, Japan) with a 40 × numerical aperture (NA = 1.4) objective.

### Statistical Analysis

All statistic data are presented as the mean ± standard error. A *p* value < 0.05 was considered to represent statistical significance. The four groups in this study were: (1) Control group, (2) CFA group, (3) EA group, and (4) TRPV1^−/−^ group (n = 10/group). Statistical significance between groups was tested using the ANOVA test, followed by a post hoc Tukey’s test (p < 0.05 was considered statistically significant). All statistical analyses were carried out using the statistical package SPSS for Windows (Version 21.0, SPSS, Chicago, Illinois, USA).

## Results

### Electroacupuncture or deletion of the TRPV1 gene significantly reduced mechanical and thermal hyperalgesia in a mouse model of inflammatory pain

**Figure 1:**
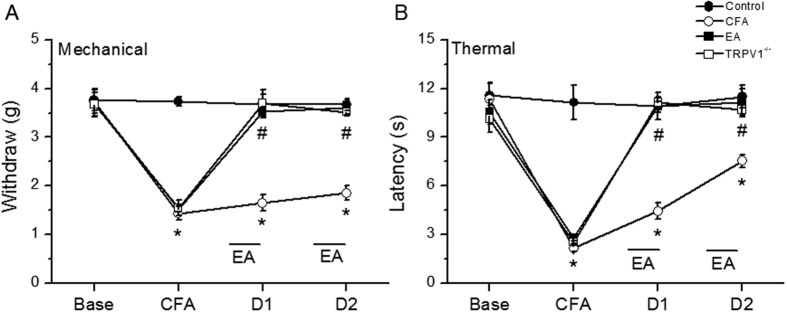
Expressions in the withdraw threshold and latency of mice in the von Frey (**A**) and radial heat test (**B**). The picture shows that analgesic effect of EA could be detected on day 1 and day 2 after treatment. *p < 0.05 means groups compared with control. ^#^p < 0.05 means groups compared with CFA group. n = 10. CFA = complete Freund’s Adjuvant; EA = Electroacupuncture; TRPV1^−/−^ = transient receptor potential V1 null mice.

Mechanical sensitivity was not different among the four groups at basal conditions (Control: 3.76 ± 0.22 g, CFA: 3.73 ± 0.24 g, EA: 3.71 ± 0.28 g, and TRPV1^−/−^: 3.67 ± 0.25 g). Injecting normal saline did not induce mechanical hyperalgesia in the control group (Control: 3.73 ± 0.1 g); however, a significantly lower pain threshold was observed in the other three groups after injecting CFA (CFA: 1.43 ± 0.14 g, EA: 1.53 ± 0.07 g, and TRPV1^−/−^: 1.55 ± 0.15 g). In addition, mechanical hyperalgesia was observed in the CFA group on days 1 and 2 after CFA injection (CFA: 1.65 ± 0.16 g and 1.85 ± 0.15 g, respectively) when compared with the other groups (Control: 3.68 ± 0.29 g and 3.67 ± 0.12 g; EA: 3.53 ± 0.09 g and 3.61 ± 0.14 g; and TRPV1^−/−^: 3.69 ± 0.19 g and 3.52 ± 0.09 g, respectively). The Hargreaves’ test showed similar results (Fig. 1B). The withdraw latencies were similar among groups prior to CFA injection (Control: 11.58 ± 0.8 s, CFA: 11.35 ± 0.94 s, EA: 10.55 ± 0.74 s, and TRPV1^−/−^: 10.14 ± 0.83 s). After CFA injection, the withdraw latency in the control group (Control: 11.15 ± 1.07 s) was significantly higher (*p* < 0.05) than the other groups (CFA: 2.16 ± 0.22 s, EA: 2.81 ± 0.19 s, and TRPV1^−/−^: 2.51 ± 0.09 s). In addition, the thermal threshold on days 1 and 2 after CFA injection was significantly lower in the CFA group (4.45 ± 0.49 s and 7.53 ± 0.4 s, respectively) (p < 0.05) when compared with the other groups (Control: 10.91 ± 0.84 s and 11.48 ± 0.69 s; EA: 10.89 ± 0.33 s and 11.15 ± 0.81 s; and TRPV1^−/−^: 11.14 ± 0.35 s and 10.69 ± 0.42 s, respectively).

### Electroacupuncture reduced inflammatory pain by reducing non-neuronal S100B and neuronal TRPV1 signaling pathways

**Figure 2:**
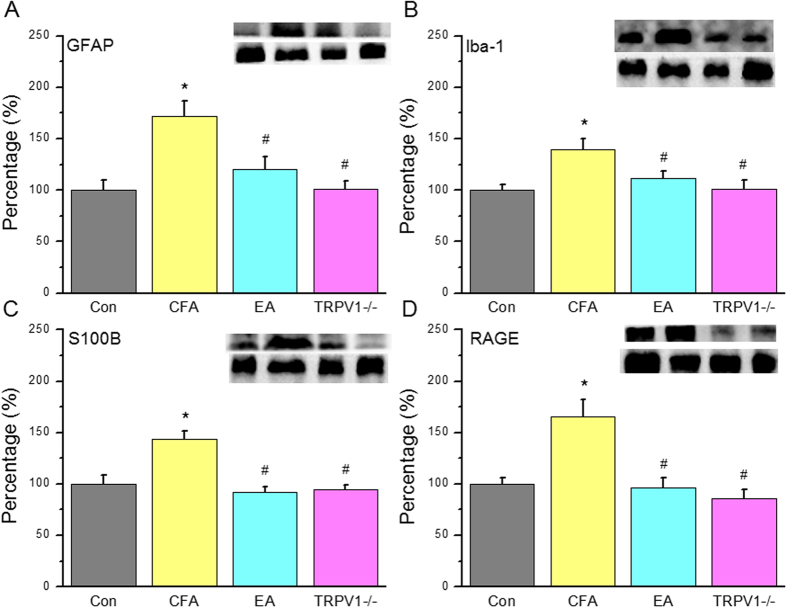
Expression levels of GFAP and associated signaling pathway proteins in DRG at day 2 after CFA injection, EA treatment and TRPV1 gene deletion. (**A**) GFAP, (**B**) Iba-1, (**C**) S100B, (**D**) RAGE. *p < 0.05 means comparison with Control group; ^#^p < 0.05 means comparison with CFA group.

We assessed GFAP and the S100B signaling pathway in our treatment model. GFAP showed a normal distribution in the control group (Fig. 2A, 100.1 ± 9.6%, n = 6) and was upregulated after CFA injection (Fig. 2A, 172.0 ± 14.5%, *p* < 0.05 compared with the Con group, n = 6). EA treatment normalized GFAP expression (Fig. 2A, 120.6 ± 11.5%, *p* < 0.05 compared with the CFA group, n = 6). A similar phenomenon was observed in the TRPV1^−/−^ group (Fig. 2A, 100.9 ± 7.9%, *p* < 0.05 compared with the CFA group, n = 6). Western blot results showed that Iba-1 (microglial cell marker) expression was increased after CFA injection (Fig. 2B, 139.6 ± 10.5%, *p* < 0.05 compared with the Con group, n = 6) and attenuated by EA (Fig. 2B, 11138 ± 7.2%, *p* < 0.05 compared with the CFA group, n = 6) and TRPV1 deletion (Fig. 2B, 100.7 ± 9.4%, *p* < 0.05 compared with the CFA group, n = 6). S100B, which is released from astrocytes, was increased in CFA groups (Fig. 2C, 143.4 ± 8.1%, *p* < 0.05, n = 6). EA (Fig. 2C, 92.2 ± 5.0%, *p* < 0.05 compared with the CFA group, n = 6) and TRPV1 deletion (Fig. 2C, 94.3 ± 4.3%, *p* < 0.05 compared with the CFA group, n = 6) significantly attenuated the increase in S100B. Tissues incubated with an antibody for the S100B receptor RAGE showed similar results (Fig. 2D, Con: 99.9 ± 5.7%, CFA: 165.7 ± 16.5%, EA: 95.9 ± 10.1%, and TRPV1^−/−^: 86.4 ± 8.6%, n = 6).

**Figure 3:**
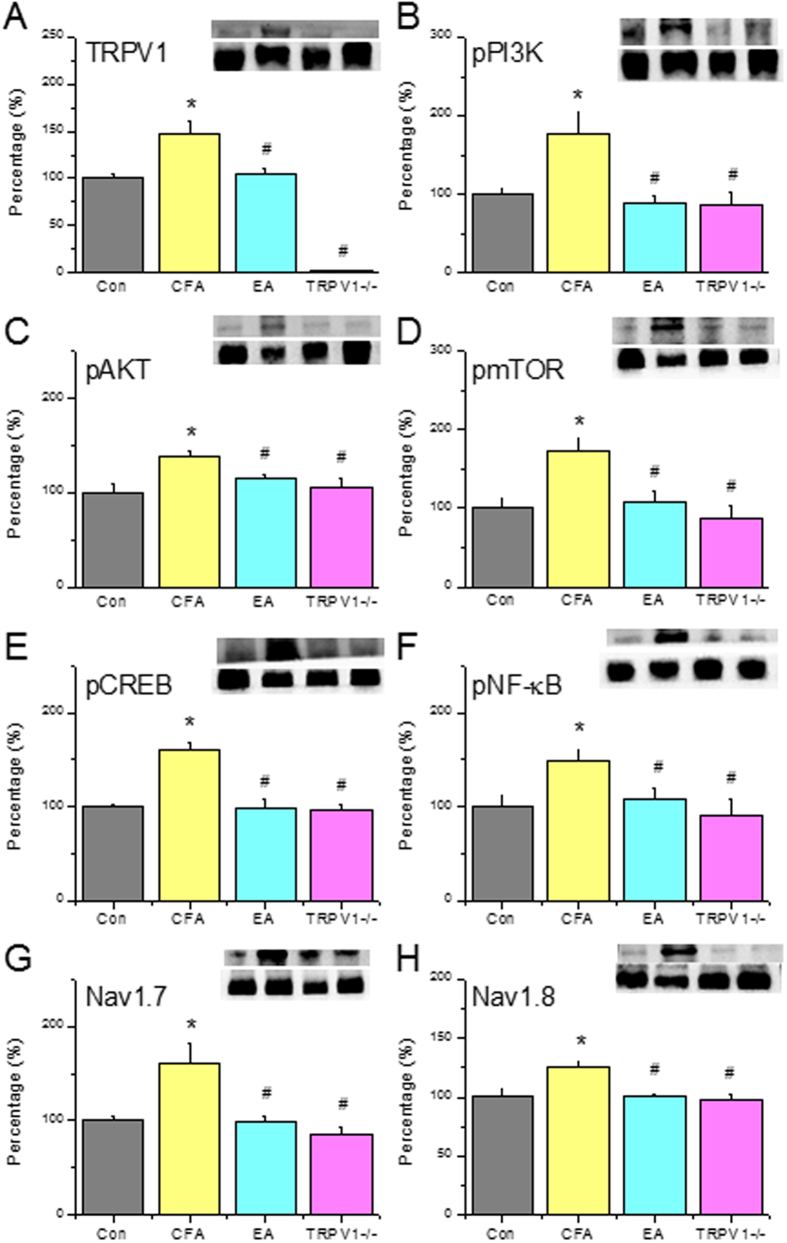
Expression levels of TRPV1 and associated signaling pathway proteins in DRG at day 2 after CFA injection, EA treatment and TRPV1 gene deletion. (**A**) TRPV1, (**B**) pPI3K, (**C**) pAkt, (**D**) pmTOR, (**E**) pCREB, (**F**) pNFκB, (**G**) Nav1.7, and (**H**) Nav1.8. *p < 0.05 means comparison with Control group; ^#^p < 0.05 means comparison with CFA group.

Next, we assessed if TRPV1 channels and related molecules are essential for the inflammatory pain response. TRPV1 was increased on day 2 after CFA injection (Fig. 3A, Control: 100.2% ± 4.9%, CFA group: 147.2% ± 14.5%, *p* < 0.05, n = 6). This increase was reversed in EA and TRPV1^−/−^ groups (Fig. 3A, EA: 104.2% ± 6.2%, *p* < 0.05 compared with the CFA group, n = 6). The increase in pPI3K observed in the CFA group (Fig. 3B, Control: 99.8% ± 7.6%, CFA group: 177.6% ± 24.5%, *p* < 0.05, n = 6) was attenuated by EA and TRPV1 deletion (Fig. 3B, EA: 89.6% ± 8.4%; TRPV1^−/−^: 85.6% ± 17.5%, all *p* < 0.05 compared with the CFA group, n = 6). Similar results were obtained for downstream molecules, including pAkt (Fig. 3C, Control: 100.3% ± 9.5%; CFA: 137.9% ± 6.7%; EA: 114.2% ± 5.1%; and TRPV1^−/−^: 106.3% ± 9.1%, n = 6) and pmTOR (Fig. 3D, Control: 100.1% ± 11.9%; CFA: 173.8% ± 16.2%; EA: 106.9% ± 13.9%; and TRPV1^−/−^: 86.3% ± 16.1%, n = 6).

Next, we assessed inflammation induced gene transcription using pCREB and pNFκB as indicators. The increase in pCREB observed in the CFA group (Fig. 3E, Control: 100.1% ± 2.5%, CFA group: 159.6% ± 9.3%, *p* < 0.05, n = 6) was normalized by EA and TRPV1 deletion (Fig. 3E, EA: 97.9% ± 10.8%; TRPV1^−/−^: 95.8% ± 6.6%, all *p* < 0.05 compared with the CFA group, n = 6). Similar data were obtained for pNFκB (Fig. 3F, Control: 100.7% ± 11.4%; CFA: 148.9% ± 12.2%; EA: 108.7% ± 11.0%; and TRPV1^−/−^: 91.5% ± 17.1%, n = 6). Furthermore, nociceptive Nav1.7 expression was increased in the CFA group (Fig. 3G, CFA group: 160.1% ± 21.1%, *p* < 0.05, n = 6). This increase was normalized by EA and TRPV1 deletion (Fig. 3G, EA: 98.7% ± 5.9%; TRPV1^−/−^: 85.1% ± 8.3%, all *p* < 0.05 compared with the CFA group, n = 6). Similar results were obtained in DRGs incubated with Nav1.8 (Fig. 3H, CFA: 126.5% ± 4.0%; EA: 100.1% ± 3.3%; and TRPV1^−/−^: 98.9% ± 4.2%, n = 6). These data suggest that the TRPV1-associated signaling pathway is essential for the inflammatory pain response in mice.

### Electroacupuncture reduced the non-neuronal S100B and neuronal TRPV1 signaling pathways in the spinal cord of inflamed mice

**Figure 4:**
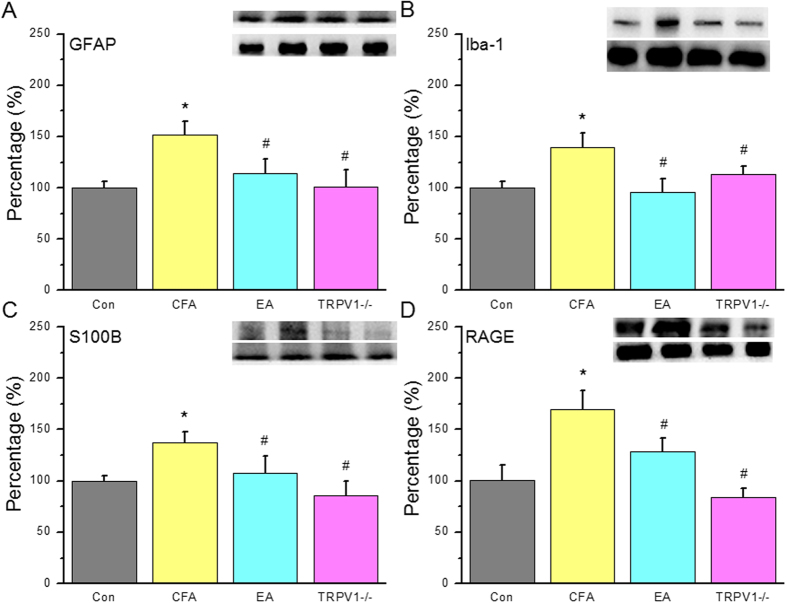
Expression levels of GFAP and associated signaling pathway proteins in SCDH after CFA injection, EA treatment and TRPV1 gene deletion. (**A**) GFAP, (**B**) Iba-1, (**C**) S100B, (**D**) RAGE. *p < 0.05 means comparison with Control group; ^#^p < 0.05 means comparison with CFA group.

To assess central sensitization effects, we examined the aforementioned molecules in SCDH tissue. GFAP was increased in the SCDH of the CFA group (Fig. 2A, 100.1 ± 6.0%, n = 6) and was upregulated after CFA injection (Fig. 4A, 151.8 ± 13.0%, *p* < 0.05 compared with the Con group, n = 6). GFAP overexpression was reduced in the EA and TRPV1^−/−^ groups (Fig. 4A, EA: 114.6 ± 13.2%, TRPV1^−/−^ 101.4 ± 16.4%, *p* < 0.05 compared with the CFA group, n = 6). Iba-1 expression was increased in CFA mice (Fig. 4B, 139.8 ± 13.7%, *p* < 0.05 compared with the Con group, n = 6), and this effect was attenuated by EA (Fig. 4B, 95.9 ± 13.3%, *p* < 0.05 compared with the CFA group, n = 6) and TRPV1 deletion (Fig. 4B, 113.7 ± 7.5%, *p* < 0.05 compared with the CFA group, n = 6). S100B was increased in CFA mice (Fig. 4C, 137.8 ± 10.2%, *p* < 0.05, n = 6), and this increase was reversed by EA (Fig. 4C, 107.6 ± 16.3%, *p* < 0.05 compared with the CFA group, n = 6) and TRPV1 deletion (Fig. 4C, 85.3 ± 14.5%, *p* < 0.05 compared with the CFA group, n = 6). Similar data were obtained for RAGE (Fig. 4D, CFA: 170.0 ± 17.9%; EA: 128.3 ± 13.4%; and TRPV1^−/−^ 84.3 ± 8.6%, n = 6).

**Figure 5:**
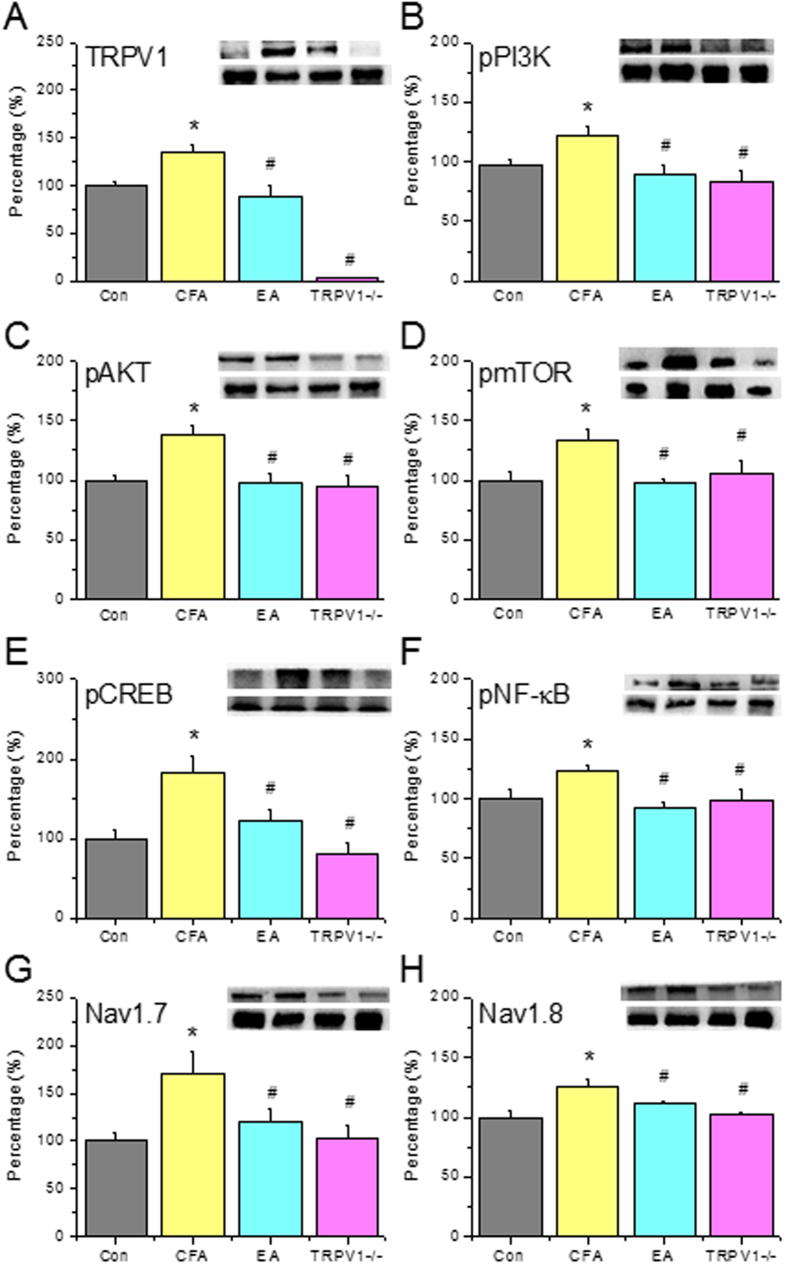
Expression levels of TRPV1 and associated signaling pathway proteins in SCDH after CFA injection, EA treatment and TRPV1 gene deletion. (**A**) TRPV1, (**B**) pPI3K, (**C**) pAkt, (**D**) pmTOR, (**E**) pCREB, (**F**) pNFκB, (**G**) Nav1.7, and (**H**) Nav1.8. *p < 0.05 means comparison with Control group; ^#^p < 0.05 means comparison with CFA group.

TRPV1 was increased in the SCDH at day 2 after CFA injection (Fig. 5A, Control: 99.6% ± 4.2%, CFA group: 135.2% ± 6.8%, *p* < 0.05, n = 6) and attenuated in EA and TRPV1^−/−^ groups (Fig. 5A, EA: 88.5% ± 11.7%, *p* < 0.05 compared with the CFA group, n = 6). pPI3K was increased in the CFA group (Fig. 5B, Control: 96.9% ± 5.0%, CFA group: 121.6% ± 7.6%, *p* < 0.05, n = 6) and attenuated in EA and TRPV1^−/−^ groups (Fig. 5B, EA: 89.2% ± 8.0%; TRPV1^−/−^: 83.1% ± 9.4%, all *p* < 0.05 compared with the CFA group, n = 6). Similar results were obtained for pAkt (Fig. 5C, Control: 99.9% ± 4.8%; CFA: 138.7% ± 6.2%; EA: 98.7% ± 7.2%; and TRPV1^−/−^: 94.6% ± 9.6%, n = 6) and pmTOR (Fig. 5D, Control: 99.9% ± 7.2%; CFA: 132.9% ± 9.5%; EA: 97.6% ± 3.8%; and TRPV1^−/−^: 106.0% ± 11.2%, n = 6). Inflammation triggered the increase of transcription factors, including pCREB (Fig. 5E, CFA: 182.7% ± 22.2%; EA: 123.2% ± 14.1%; and TRPV1^−/−^: 81.1% ± 13.9%, n = 6) and pNFκB (Fig. 5F, CFA: 123.2% ± 5.1%; EA: 92.5% ± 5.0%; and TRPV1^−/−^: 98.5% ± 9.3%, n = 6). Moreover, nociceptive Nav1.7 was increased in the CFA group (Fig. 5G, CFA group: 171.3% ± 23.1%, *p* < 0.05, n = 6), and this effect was reversed in EA and TRPV1^−/−^ groups (Fig. 5G, EA: 119.9% ± 13.1%; TRPV1^−/−^: 103.6% ± 11.7%, all *p* < 0.05 compared with the CFA group, n = 6). Similar results were obtained in SCDH tissue incubated with Nav1.8 (Fig. 5H, Control: 99.9% ± 5.2%; CFA: 126.2% ± 6.0%; EA: 111.1% ± 2.5%; and TRPV1^−/−^: 102.2% ± 2.0%, n = 6). These data suggest that the TRPV1-associated signaling pathway is essential for central sensitization at the SC level.

### CFA-induced increases in nociceptive Nav1.7 and Nav1.8 immunoreactive signals were attenuated by EA and TRPV1 gene deletion

**Figure 6:**
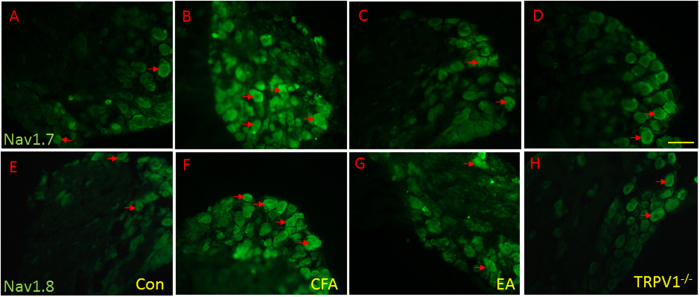
Expressions of Nav1.7, and Nav1.8 in the DRG of Con, CFA, EA, and TRPV1^−/−^ mice. Nav1.7-positive neurons (green) in the DRG of (**A**) Con, (**B**) CFA, (**C**) EA, and (**D**) TRPV1^−/−^ mice. Nav1.8-positive neurons (green) in the DRG of (**E**) Con, (**F**) CFA, (**G**) EA, and (**H**) TRPV1^−/−^ mice. Scale bar means 100 μm.

Immunohistochemical staining visualized by green fluorescence showed that Nav1.7 was expressed in DRG neurons. Nav1.7 expression was increased after CFA injection, and further this effect was attenuated in EA and TRPV1^−/−^ groups (Fig. 6A–D). Nociceptive Nav1.8-positive DRG neurons were increased in the CFA group, and this effect was reversed in EA and TRPV1^−/−^ groups (Fig. 6E–H).

### Injecting the PI3K inhibitor LY294002 significantly reduces inflammatory pain and pAkt-pmTOR protein level

**Figure 7:**
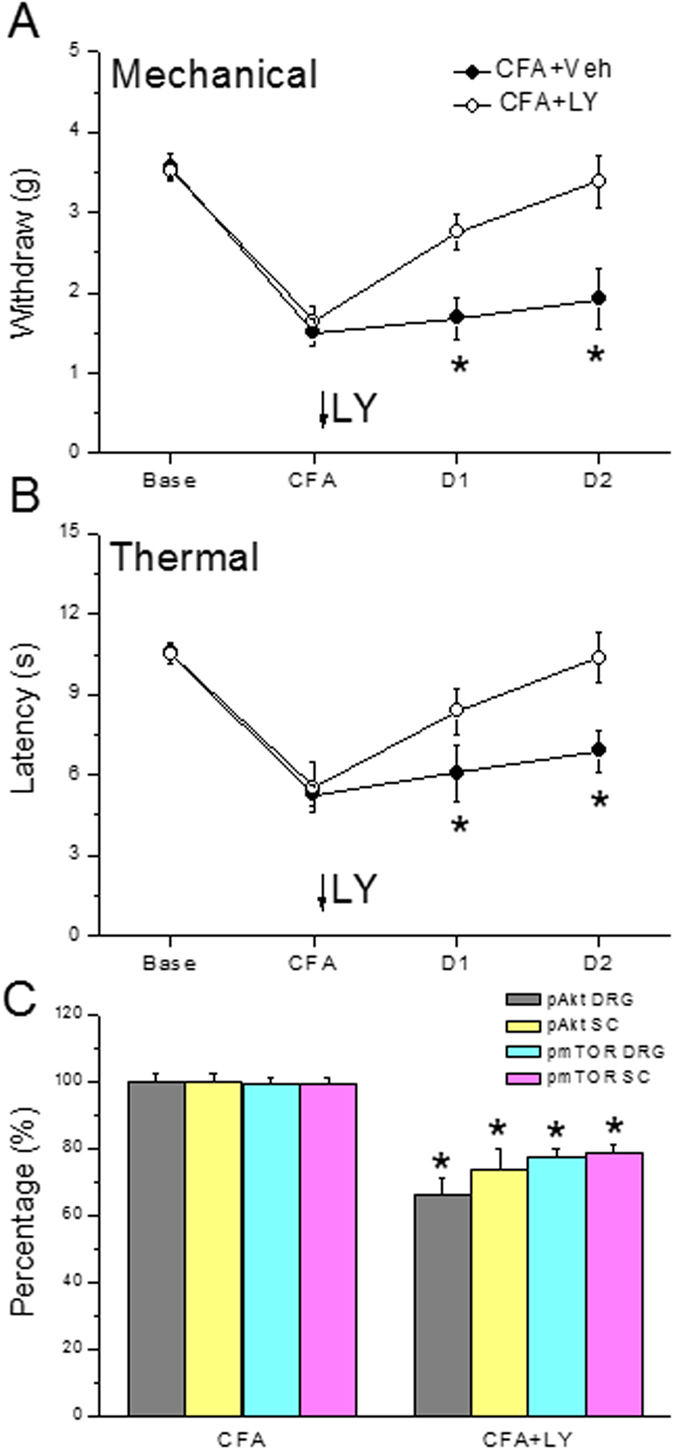
Expression of the withdrawal threshold and latency of mice in the von Frey test (**A**), radial heat test (**B**), and pAkt and pmTOR protein expression (**C**). The photograph shows that the analgesic effect of LY294002 was detectable on days 1 and 2 after treatment. *p < 0.05 vs. CFA. n = 10. CFA = complete Freund’s Adjuvant; LY = LY294002.

Mechanical sensitivity did not differ between the two groups at baseline (Fig. 7A, CFA: 3.58 ± 0.17 g and LY: 3.52 ± 0.1 g). A significantly lower pain threshold was observed in the two groups after injecting CFA (Fig. 7A, CFA: 1.51 ± 0.17 g and LY294002: 1.64 ± 0.18 g). In addition, mechanical hyperalgesia was observed in the CFA group on days 1 and 2 after the CFA injection (Fig. 7A, CFA: 1.68 ± 0.26 g and 1.92 ± 0.37 g, respectively) compared with that in the LY294002 group (Fig. 7A, LY294002: 2.76 ± 0.22 g and 3.39 ± 0.32 g). The Hargreaves’ test showed similar results (Fig. 7B). Withdrawal latencies were similar between the two groups prior to the CFA injection (Fig. 7B, CFA: 10.58 ± 0.27 s and LY294002: 10.52 ± 0.39 s), and withdrawal latency decreased in both groups after the CFA injection (Fig. 7B, Control: 5.26 ± 0.39 s and LY294002: 5.53 ± 0.91 s). In addition, the thermal threshold on days 1 and 2 after the CFA injection was significantly lower in the CFA group (Fig. 7B, 6.07 ± 1.04 s and 6.89 ± 0.79 s, respectively) (p < 0.05), compared with that in the LY294002 group (Fig. 7B, 8.37 ± 0.89 s and 10.38 ± 0.95 s, respectively). The PI3K inhibitor LY294002 significantly attenuated activation of pAkt and pmTOR in the DRG and SC, suggesting a relationship with PI3K, pAkt and pmTOR (Fig. 7C).

### Opioid and adenosine A1 receptors are critical for electroacupuncture-mediated analgesia in a mouse model of inflammatory pain

**Figure 8:**
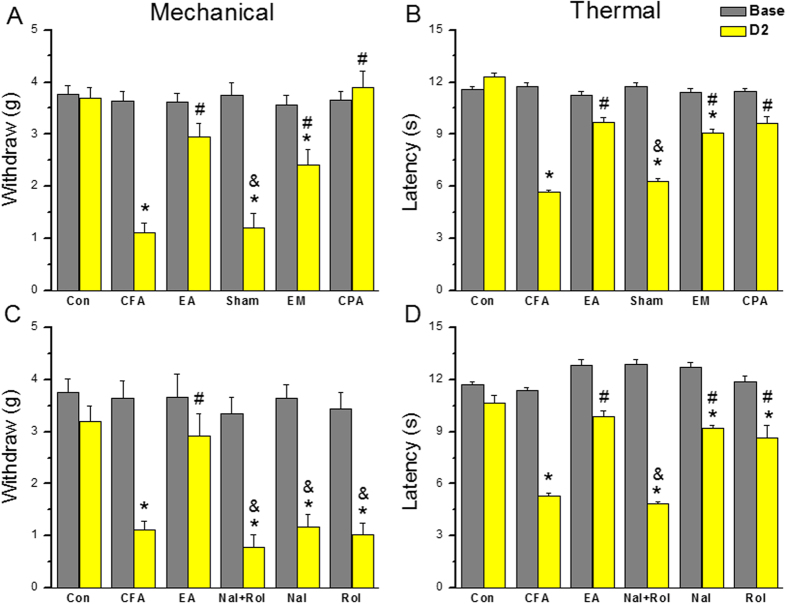
Opioid and adenosine receptor agonist administration relieved mechanical and thermal pain. Con: saline-injected control; CFA: CFA-induced inflammation; EA: EA at the ST36 acupoint; Sham: EA at the nonacupoint; EM: endomorphin; CPA: N^6^-Cyclopentyladenosine. *p < 0.05 means comparison with Control group; ^#^p < 0.05 means comparison with CFA group; ^&^p < 0.05 means comparison with EA group.

We next determined how EA relieves inflammatory pain. EA at acupoint ST36 significantly reduced CFA-induced mechanical hyperalgesia (Fig. 8A, 2.9 ± 0.3 g, *p* < 0.05 compared with CFA inflammation group, n = 8). This effect was not observed in the sham control (Fig. 8A1.2 ± 0.3 g, *p* > 0.05 compared to CFA inflammation group, n = 8). An i.p. injection of the opioid-specific agonist EM partially reduced mechanical hyperalgesia (Fig. 8A, 2.4 ± 0.3 g, *p* < 0.05 compared with the CFA group, n = 8). An i.m. injection of the adenosine receptor agonist CPA into acupoint ST36 reduced mechanical hyperalgesia (Fig. 8A, 3.9 ± 0.3 g, *p* < 0.05 compared with the CFA group, n = 8). Similar results were observed for thermal hyperalgesia. EA significantly attenuated thermal hyperalgesia (Fig. 8B, 9.7 ± 0.2 s, *p* < 0.05 compared with the CFA group, n = 8), and this effect was not observed in the sham group (Fig. 8B, 6.3 ± 0.2 s, *p* > 0.05 compared with the CFA group, n = 8). The administration of EM (Fig. 8B, 9.1 ± 0.2 s, *p* < 0.05 compared with the CFA group, n = 8) and CPA (Fig. 8B, 9.6 ± 0.4 s, *p* < 0.05 compared with the CFA group, n = 8) significantly reduced thermal hyperalgesia in a mouse model of inflammatory pain.

We administered an i.p. injection of naloxone, a μ-opioid receptor antagonist intraperitoneally, and/or an i.m. injection of rolofylline, an adenosine A1 receptor antagonist, into ST36 acupoint to determine if the analgesic effects of EA can be blocked. Mechanical hyperalgesia was restored after the co-injection of naloxone and rolofylline (Fig. 8C, 0.8 ± 0.2 g, *p* < 0.05 compared with the EA group, n = 8). Mechanical hyperalgesia was also observed in mice treated with naloxone (Fig. 8C, 1.2 ± 0.2 g, *p* < 0.05 compared with the EA group, n = 8) or rolofylline alone (Fig. 8C, 1.0 ± 0.2 g, *p* < 0.05 compared with the EA group, n = 8). In terms of thermal hyperalgesia, the co-injection of naloxone and rolofylline significantly reversed the analgesic effects of EA (Fig. 8D, 4.8 ± 0.1 s, *p* < 0.05 compared with the EA group, n = 8). A single injection of naloxone or rolofylline alone also suppressed EA-mediated analgesia (Fig. 8A, Nal: 9.2 ± 0.2 s, Rol: 8.6 ± 0.7 s, *p* < 0.05 compared with the CFA group, n = 8).

### Electroacupuncture, endomorphin, and rolofylline significantly reduced CFA-induced inflammatory pain via Nav1.8 downregulation

**Figure 9:**
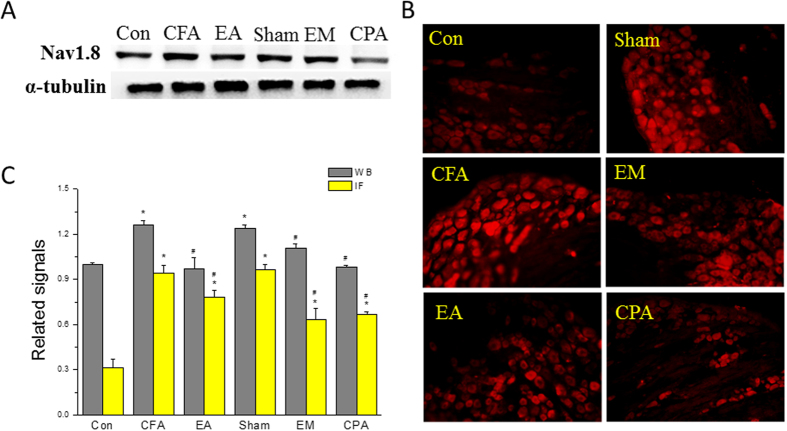
Nav1.8 expressions in DRGs by using Western and immunofluorescence staining. (**A**) DRGs lysates were immunoreactive with antibodies to Nav1.8 and α-tubulin. (**B**) DRG slices were immunoreactive with antibodies to Nav1.8 (red). (**C**) The expression of Nav1.8 was analyzed and plotted. *p < 0.05, as compared to control group, ^#^*p* < 0.05, as compared to CFA inflammation group.

We analyzed the effect of EA analgesia on Nav1.8 expression. Western blotting results showed that Nav1.8 was increased in CFA mice (Fig. 9A, 126.4 ± 2.5%, *p* < 0.05 compared with the Con group, n = 6), and this increase was abolished in the EA group (Fig. 9A, 97.1 ± 7.6%, *p* < 0.05 compared with the CFA group, n = 6) but not the sham group (Fig. 9A, 123.7 ± 13.7%, *p* < 0.05 compared with the EA group, n = 6). Interestingly, an EM injection reduced the overexpression of Nav1.8 (Fig. 9A, 110.6% ± 3.3%, n = 6). A similar result was observed in the CPA group (Fig. 9A98.3% ± 1.1%, n = 6). Immunofluorescence staining confirmed an increase in Nav1.8-immunoreactivity in the CFA group. This effect was reduced in the EA, EM, and CPA groups (Fig. 9B, n = 6). All of the results were analyzed and plotted (Fig. 9C).

## Discussion

In our current study, we confirmed that a CFA injection successfully evokes inflammatory pain in mice. The mechanical and thermal hyperalgesia observed in the CFA-induced inflammatory pain model were attenuated on days 1 and 2 by EA stimulation at bilateral ST36 acupoints and TRPV1 gene deletion. We also showed that astrocytes, microglia, S100B, and the TRPV1-related signaling pathway were essential for EA analgesia. EA reduced both mechanical and thermal hyperalgesia via the activation of opioid and adenosine A1 receptors and downregulation of Nav1.8 expression.

A recent study showed that transient hyperalgesia can be partially induced via the acid-sensing ion channel 3 pathway even with TRPV1 gene deletion^19^. However, TRPV1 gradually suppresses pain, which suggests that the TRPV1 pathway is essential but is not the only inflammatory pain pathway. S100B in the DRG and SCDH was increased on day 2 after CFA injection. S100B is mainly secreted by astrocytes^20^,^21^ and stimulates RAGE on neurons and microglia, thus activating these cell types. Using the microglia/macrophage-specific calcium-binding protein Iba-1, we determined that the number of microglia in the DRG and SCDH was increased after CFA injection. Previous studies showed that activated microglia secrete IL-1^22^, IL-6^22^, and TNF-α^22^,^23^. Our data showed that TRPV1^12^,^24^, PI3K^12^,^25^, AKT^12^,^25^, mTOR, CREB^12^,^26^, NF-κB, Nav1.7^2^,^12^, and Nav1.8^2^,^12^ were increased after CFA injection. Furthermore, the number of Nav1.7 and Nav1.8 channels was increased in DRG neurons. Thus, the intraplantar injection of CFA successfully induced peripheral nociceptive neurons to generate trains of action potentials toward the DRG and SCDH. Next, the neurons and microglia cells of the spinal DRG and SCDH are activated, thus producing mechanical and thermal hyperalgesia. The increased expression of the aforementioned molecules within neurons and microglia was attenuated by EA and TRPV1 gene deletion. To the best of our knowledge, very few studies have explored the effects of EA on inflammation-activated microglia. Our data show that EA suppresses the activation of microglia in an inflammatory pain model.

Zhang *et al*. reported that EA at a frequency of 10 Hz significantly reduced CFA-induced hind paw edema. Moreover, EA attenuated the inflammatory response via the hypothalamus-pituitary-adrenal axis (HPA) and nervous system^27^. Recently, EA was shown to suppress the inflammation-induced expression of neurokinin-1 in the SCDH of rats^28^. These phenomena were not observed in sham groups, which suggests acupoint-specific effects^27^,^28^. Numerous studies have investigated the role of different Nav channels in pain, neuron excitability, and action potential firing^29^,^30^. Nav1.7 was greatly expressed in C-fiber free nerve endings and played a crucial role in the propagation of nociceptive information^31^. Recent studies utilizing antisense and knockout mice strongly support the use of Navs as analgesic drugs^32^,^33^. Derivatives from benzazepinone and imidazopyridine were developed to block Nav1.7 channels for pain treatment^34^. Our results clearly indicate that EA reliably attenuated CFA-induced inflammatory pain by ameliorating Nav1.7 overexpression.

The chronic intrathecal administration of Nav1.8 antisense successfully attenuated Nav1.8-induced currents and decreased mechanical allodynia after intraplantar CFA injections^35^. Specific Nav channel blockers are a potentially useful treatment for inflammatory pain. A-803467, a novel and specific blocker of the Nav1.8 channel, ameliorated inflammatory pain in rats^36^. Thus, our investigation into inflammatory pain using ancient acupuncture mechanisms is highly valuable and our findings can be further applied to clinical medicine. EA and the administration of an adenosine A1 receptor agonist at ST36 attenuated pain behaviors, whereas endomorphin 1 administration at ST36 did not (data not shown). This finding suggests that endomorphin 1 may work in the CNS but not at acupoints, which is supported by a previous study^37^. We injected EM intraperitoneally and showed that Nav1.8 overexpression was reversed. A similar result was observed in the CPA (local injection) group. Activation of opioid receptors modulates peripheral inflammation in local tissues^38^ and suppresses Ca^2+^ and/or Na^+^ currents in primary afferent neurons^39^. Stein and Kuchler reported that activating opioid receptors further activates the Gi protein to inhibit TRPV1^40^.

PKA^41^ and PKCε^42^ phosphorylate Nav1.8, increase Nav1.8 currents, lower the threshold voltage for activation, and produce a depolarizing shift. Inflammatory cytokines, such as interleukin-1β (IL-1β)^43^ and IL-6^44^, activate astrocytes and mediate neuroprotection in a PKCε-dependent manner^45^,^46^. Our immunostaining data showed that Nav1.8 was increased after CFA-induced inflammation. This effect was ameliorated by EA and the activation of opioid and adenosine receptors. Furthermore, opioid and adenosine antagonists significantly blocked the analgesic effects of EA. Kim *et al*. suggested that PI3K and Akt phosphorylation increases in response to carrageenan-induced inflammatory pain and is abolished by EA. Furthermore, the PI3K inhibitor significantly attenuates carrageenan-induced hyperalgesia^47^. Xu *et al*. reported that activation of pPI3K-Akt in the rat DRG and SC is crucial for neuropathic pain after spinal nerve ligation. An intrathecal injection of a PI3K or Akt inhibitor reduces neuropathic pain behavior. Immunohistochemistry indicated that an intrathecal injection of the PI3K inhibitor significantly inhibits activation of Akt in L5 of the DRG and SC^48^. Sun *et al*. showed that Akt, which is distributed mainly in small to medium DRG, increases in DRG 5 min after an intradermal injection of capsaicin (TRPV1 activator). TRPV1 is also double labeled with Akt. Behavioral tests suggested that an intradermal injection of the PI3K inhibitor was significantly inhibited by capsaicin-induced hyperalgesia. A similar result was observed with an Akt inhibitor^49^. Xu *et al*. reported that pretreatment with a PI3K inhibitor prevents activation of Akt in a dose-dependent manner in mice with plantar incision-induced pain. Inhibiting spinal PI3K further prevents pain behavior and c-Fos expression^50^. Kay *et al*. suggested that suppressing endogenous PI3K/Akt activity with the potent PI3K inhibitor LY294002 abolishes pCREB in mice with visceral inflammation-induced cystitis^51^. Our results showed that injecting the PI3K inhibitor significantly reduces inflammatory pain and pAkt-pmTOR protein level.

## Conclusion

**Figure 10:**
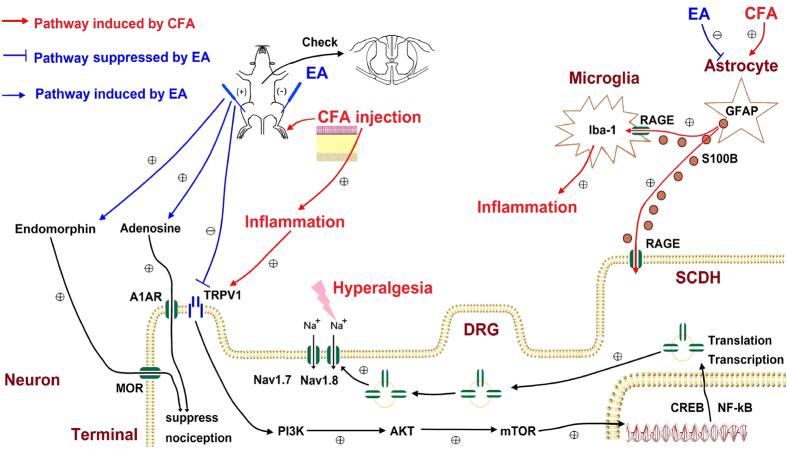
Schematic illustration of neuronal and non-neuronal mechanisms in EA-mediated analgesia of CFA-induced inflammatory pain. Summary diagram of how astrocyte, glial cells, and TRPV1 is crucial for inflammatory pain and related mechanisms. Our results show that EA can reduce S100B release from non-neuronal cell. We also indicated that EA can trigger the release of opioid and adenosine receptors for relieving inflammatory pain through TRPV1 pathway.

CFA induced mechanical and thermal hyperalgesia in mice. This effect was accompanied by the activation of astrocytes and glial cells, which then released the RAGE ligand S100B. Inflammation also activated TRPV1 and triggered a signaling pathway that activated nociceptive Nav channels. These phenomena were abolished by EA and deletion of the TRPV1 gene. We demonstrated that EA triggers the release of both endogenous opiates and adenosine to relieve inflammatory pain in mice. This study identified a potential TRPV1-mediated signaling mechanism (Fig. 10). Given the specific analgesic effect of EA on inflammatory hyperalgesia, TRPV1 antagonists could further translate into clinical efficacy for treating pain. Our findings offer insight for EA clinical trials in patients with inflammatory pain.

## Additional Information

**How to cite this article**: Liao, H.-Y. *et al*. Electroacupuncture Attenuates CFA-induced Inflammatory Pain by suppressing Nav1.8 through S100B, TRPV1, Opioid, and Adenosine Pathways in Mice. *Sci. Rep.* **7**, 42531; doi: 10.1038/srep42531 (2017).

**Publisher's note:** Springer Nature remains neutral with regard to jurisdictional claims in published maps and institutional affiliations.
